# Management of arterial hypertension: Challenges and opportunities

**DOI:** 10.1002/clc.23938

**Published:** 2022-11-24

**Authors:** Beatriz V. Silva, Catarina Sousa, Daniel Caldeira, Ana Abreu, Fausto J. Pinto

**Affiliations:** ^1^ Centro Cardiovascular da Universidade de Lisboa – CCUL, CAML, Faculdade de Medicina, Universidade de Lisboa Lisboa Portugal; ^2^ Serviço de Cardiologia Hospital Universitário de Santa Maria – CHULN Lisboa Portugal

## 1 INTRODUCTION

Systemic arterial hypertension (HTN) is still the leading cause of disability‐adjusted life‐years and death globally.^1^ It affects over one billion adults globally and 10% of the global healthcare spending is directly related to HTN and its complications.^2^ The progress in HTN awareness, treatment, and control rates has plateaued or even deteriorated in the last 20 years, particularly in low‐ and middle‐income countries.^3^ The improvement of HTN in low‐ and middle‐income countries face significant resource constraints, governance difficulties, lack of funding and poor prioritization due to competing priorities such as infectious diseases and maternal and child health.^4^, ^5^ Considering the burden of HTN, improving its detection, management and control deserves high priority. The World Heart Federation (WHF) developed a roadmap for HTN, advising health system policies and clinical practices as part of its commitment to improving global cardiovascular health.^5^

In this paper, we will briefly discuss some barriers and their potential solutions to improve prevention, detection, and management of HTN.

## 2 PREVENTION: IMPACT OF NONPHARMACOLOGICAL INTERVENTIONS

Nonpharmacological interventions are an essential component for the prevention and management of HTN. The recommended lifestyle measures shown to reduce blood pressure (BP) include weight management, a healthy diet, salt restriction, regular physical activity and moderation of alcohol consumption.^3^ Unfortunately, only a minority of adults change their lifestyle after a diagnosis of HTN, and sustainability is difficult, posing a substantial challenge for the successful implementation of a healthy lifestyle.^4^ Thus, community‐level strategies that accelerate the implementation of health‐promoting policies can create an environment where people are more compelled to adopt or continue healthy behaviors and may have the greatest influence on health outcomes.^5^ It is also important to stress that this education should be done at all age strata and that children should not be forgotten because they are the adults of the future and influencers of current adults. Starting at young age (at home or school) the adoption of a healthy lifestyle is much easier than changing old habits in adults.

### 2.1 Diet

Sodium restriction in the diet has been shown to have a BP‐lowering effect and reduce cardiovascular disease risk.^6^ Effective sodium restriction may reduce the number or dosage of antihypertensive drugs necessary to achieve BP control in people with HTN.^6^, ^7^ WHO recommends reducing sodium intake to <2 g/day (5 g/day salt) in adults.^5^ Therefore, reduction of salt intake should be a public health priority, requiring combined effort between government policies, food industry and population education.^3^

Salt substitution is also a nonpharmacological approach to reduce BP.^8^ It involves the partial replacement of sodium chloride with any combination of other salt containing potassium, magnesium, or aluminum. A salt substitute can reduce systolic and diastolic BP.^9^ Consequently, salt replacement strategy can halve the incidence of HTN in individuals without previous HTN and it may also reduce the incidence of cardiovascular events.^8^, ^9^

In general, the population should be advised to eat a healthy balanced diet containing vegetables, fresh fruit, fish, whole grains, low‐fat products, and unsaturated fatty acids, and reduce consumption of refined sugar, saturated fat, and cholesterol.^5^, ^7^ In individuals with HTN, increasing potassium intake lowers BP, especially among those with a diet with a high intake of sodium,^6^ therefore decreasing the risk of cardiovascular disease. The aim is for 3500–5000 mg per day, preferably by a diet rich in potassium, if there are no contra‐indications, namely the presence of high plasmatic levels of potassium.

The modified Dietary Approaches to Stop Hypertension (DASH) diet was a potentially effective treatment for pre‐hypertensive and hypertensive patients, with an expected effect of up to 11 mmHg reduction in systolic BP.^10^ A systematic review demonstrated that the DASH diet could reduce BP, waist circumference and triglyceride concentration in hypertensive patients.^7^ The combination of low sodium intake and the DASH diet provides substantially greater BP reduction than sodium restriction or DASH diet alone.^9^, ^11^ The Mediterranean diet has shown to have cardiovascular protective effects and it can be explained at least partially by its BP lowering effects. Supporting the recommendation of a Mediterranean diet, the SUN cohort showed that a systematic adoption of this regimen led to a reduction of systolic and diastolic BP in 3.1 and 1.9 mmHg, respectively.^12^

### 2.2 Weight reduction

Excessive weight gain is associated with HTN, and obesity is closely related to conditions that can be secondary causes of HTN such as obstructive sleep apnea and/or the frequent use of nonsteroidal anti‐inflammatory drugs due to overweight‐related early arthritis. Reducing weight towards an ideal body weight decreases BP. Weight loss also improves the efficacy of antihypertensive medications.^7^ Weight loss should be achieved through a multidisciplinary approach, including dietary advice, motivational counseling, and regular exercise. In patients with overweight or obesity, a reduction of weight in 10 kg is associated with a decrease of systolic BP of 6 and 4.6 mmHg in diastolic BP.^10^

### 2.3 Regular physical activity

Regular physical activity is beneficial for preventing and treating HTN and is associated with lower cardiovascular risk and mortality.^13^ Healthy adults get additional benefits with a gradual increase in aerobic physical activity to 300 min a week of moderate‐intensity or 150 min a week of vigorous‐intensity aerobic physical activity, or an equivalent combination.^14^ A systematic review demonstrated that high BP patients who participated in any level of physical activity had a reduced CV mortality risk (16%–67%), whereas a greater than twofold increase in mortality risk was noted in nonactive individuals. Regular physical activity of low intensity and duration lowers BP less than moderate‐ or high‐intensity training, but still associates with mortality decrease.^15^ A meta‐analysis of RCTs analyzed different types of exercise and showed that aerobic endurance training, dynamic resistance training, and isometric training reduced resting SBP and DBP in general populations, whereas combined training lowers only DBP. Data suggest that isometric resistance training has the potential for the largest reductions in SBP.^16^ It is suggested that hypertensive patients should be advised to participate in at least 30 min of moderate‐intensity dynamic aerobic exercise 5–7 days per week, being resistance exercise 2–3 days per week is also advised.^7^

Initiatives such as “*Million Hearts”* recommend that communities make changes in the environment in which people live, learn and work, and create activity‐friendly routes to everyday destinations, including worksites and schools, to promote safety and convenience to enhance physical activity. Physical activity and exercise programmes need to be appealing to attract healthy and hypertensive individuals.

### 2.4 Moderation in alcohol intake

There is a positive linear association between alcohol consumption and HTN.^17^ A complete abstinence or alcohol intake limitation to ≤2 standard drinks per day with 2 days off per week is recommended and expected to reduce up to 3–4 mmHg in systolic BP.^18^ The binge drinking pattern even with a reduction of weekly alcohol consumption frequency is associated with a higher risk of HTN.^19^

### 2.5 Tobacco control programs

Reducing tobacco use by implementing effective tobacco control programs is essential to improve the cardiovascular burden of disease. Education for tobacco addiction and negative effects should start in early ages with school programs. Tobacco taxation and prices, banning tobacco advertising, prohibition of smoking in public spaces and promoting awareness of the risks of tobacco use by including health warnings on packaging and antismoking advertising, are important measures to reduce the burden of tobacco use.^20^

### 2.6 Environmental factors

Several environmental factors affect BP, such as low greenness, poor housing conditions, use of cooking fuel and indoor air pollution.^21^, ^22^ There is an association between air pollution and cardiovascular disease, and fine particulate matter <2.5 μg (PM2.5) was identified as a significant contributor to HTN.^23^ Thus, controlling indoor and outdoor air pollution, improving greenness levels, and subsiding cleaner cooking fuels may have beneficial effects on BP levels. Trials with air cleaners have shown to decrease systolic BP up to 4 mmHg. Public policies that promote a healthy environment need to be prioritized as they can play a crucial role in preventing and controlling HTN.

### 2.7 Stress and depression

Several studies suggest that prolonged stress may predispose people and animals to prolonged HTN and certain populations are at risk for the development of stress‐induced HTN. It is likely that prolonged stress‐induced HTN is the result of neurohormonal trophic factors for hypertrophy or atherosclerosis. Relaxation techniques are being used increasingly in the treatment of patients with HTN.^24^

Depression is a common feature in patients experiencing uncontrolled HTN, which may contribute to poor control. Screening for depression in hypertensive patients is a simple and cost‐effective tool that may improve outcomes and should be performed in all hypertensive patients.^25^

## 3 DIAGNOSIS AND EVALUATION

The asymptomatic nature of HTN in conjunction with its disease burden underlines the importance of routine BP screening. According to WHF, HTN screening should occur from the age of 18 and be repeated every 2 years, depending on the availability of resources, while guidelines have taken a more pragmatic approach of recommending repeat measurement between 1 and 5 years depending on BP.^7^ Opportunistic screening for HTN and frequent measurement of BP is particularly relevant in high‐risk individuals to increase awareness of HTN and for early diagnosis and initiation of appropriate treatment. In addition, evidence suggests that setting‐based screening programs improve the diagnosis and control rate of HTN.^26^ It should not be forgotten to exclude causes for secondary of HTN, which can be managed, as iatrogenic (corticotherapy, NSAID, and vasoconstrictors), renal, cardiovascular, endocrine diseases, or others.

Different equipment and techniques are used for BP measurement and the accuracy of these devices varies widely and reduces the efficacy of mass BP screening programmes to diagnose HTN accurately.^5^ Particularly in low‐resource settings, access and procurement of validated and accurate BP machines remain a challenge.^27^ In agreement with the WHO quality standard for BP machines, having enough calibrated and validated machines available to ensure screening and diagnosis should be a priority.^5^

Out‐of‐office BP measurements—such as self‐home BP monitoring (HBPM) or 24‐h ambulatory BP monitoring (ABPM)—should be encouraged and are the recommended strategy for diagnosing and managing HTN.^3^, ^7^ It also offers the advantage of encouraging patient involvement in HTN management and facilitating telemonitoring. Effective and sustainable strategies for implementing out‐of‐office BP testing need to be developed to strengthen HTN control initiatives.^5^ An important complement is to train patients and nonphysician health workers for accurate measurement of BP.^27^ Community‐based nonphysician or self‐screening could lead to the identification of new cases of HTN.^28^

Real‐life examples such as the “May Measurement Month” (MMM), an initiative led by the International Society of Hypertension and endorsed by the World Hypertension League and WHF, is an annual globally synchronized BP screening campaign designed primarily to raise awareness of the importance of BP measurement at the population level and in the process, detect untreated or inadequately treat HTN.^29^ One critical finding of MMM in 2019 was that nearly one third of the screeners had never had their BP measured previously.^29^

## 4 MANAGING HYPERTENSION

### 4.1 The role of primary care physicians

Primary care physicians and other allied healthcare workers should be at the forefront of managing HTN.^5^ The paradigm shift to move the gravity center of HTN programmes from specialized secondary care to the community levels (primary care) is essential for population‐wide impact.

### 4.2 Task sharing and shifting and team‐based care

In most countries, primary care physicians are encouraged to be the main healthcare providers regarding the prevention and control of cardiovascular risk factors. However, most of low‐income countries have an inadequate number of physicians, especially in rural and remote regions.^30^ Task‐sharing and shifting strategies that involve transferring less skilled tasks to nonphysician health workers under the supervision of a physician are effective in addressing some of the critical barriers to improve HTN control.^5^ One of the examples of having health promotion habits endorsed by trusted community members was the black barbershop trial where healthcare evaluations and prescriptions were dislocated from healthcare facilities to the community and resulted in a large reduction of systolic BP (about 20 mmHg) compared with an active encouragement of lifestyle modification and doctor appointments by barbers.^31^

Also in some countries, preventive CV multidisciplinary programs were created for the evaluation and managing CV Risk, including risk factors treatment, as in the case of HTN. Many times HTN is not alone and associates with dyslipidemia, obesity, diabetes or smoking, which increases exponentially CV risk. Metabolic syndrome is a very high‐risk situation and patients need not only HTN, which is present in 80% of cases, to be corrected, but all the other risk factors involved.^32^ HTN management should be integrated in global CV risk control.^7^

### 4.3 Pharmacological interventions

Pharmacological treatment of HTN plays a central role in its management.^7^ Several drugs have been developed and proven effective in the treatment of HTN.^7^ However, the use of antihypertensive medication differs widely by world region.^33^ Overall, the use of antihypertensive medication is more common in high‐income versus middle‐ or low‐income regions (56% vs. 29%). The Prospective Urban Rural Epidemiology (PURE) study showed that overall control was worst in low‐income and lower‐middle‐income countries (11%), compared with 19% in high‐income countries and 16% in upper‐middle‐income countries. Some reasons for the low frequency of treatment and control of HTN included insufficient therapy and poor access to the healthcare system.^5^

HTN guidelines support low‐dose pharmacological therapy initially and subsequent up‐titration and/or addition of new drugs based on the achieved BP and tolerability, as low‐dose combinations of antihypertensive agents are more effective in BP‐lowering and well‐tolerated than high‐dose monotherapies.^7^ A single‐pill combination of drugs at lower doses is recommended as it may provide improvements in adherence, efficacy, and tolerability of therapy, and most of the patients will require two or more drugs to achieve de BP targets.^7^ In 2019, single‐pill antihypertensive drug combinations were listed on the WHO Essential Medicine list.^34^ Nowadays single‐pill combinations exist with two or three antihypertensive agents.

Clinician therapeutic inertia is a contributing factor for failure to achieve BP targets.^35^ Prescription of a fixed‐dose combination when initiating antihypertensive treatment may overcome therapeutic inertia as it can reduce the number of follow‐up visits needed and has been demonstrated to achieve a higher rate of BP control.^5^, ^7^, ^35^

Inadequate supplies of essential antihypertensive drugs are a major barrier to the delivery of optimal care for the management of HTN in low‐resource settings.^5^ Relying on simple treatment protocols with specific drugs and doses at each step is recommended to facilitate the procurement of large volumes of medicines of choice, which simplifies the supply chain and lower prices.

Another important aspect relates to low adherence to anti‐hypertensive medication, which remains a major contributing factor to uncontrolled BP.^36^ Barriers to achieve high medical adherence are multifactorial and include complex medication regimens, convenience factors (e.g., dosing and frequency), forgetfulness, issues with treatment of asymptomatic disease (including fear of possible or experienced adverse events and perceived lack of treatment benefit), cost of treatment and limited access to care.^36^, ^37^

Besides pharmacological treatment for HTN, we should not forget that the pharmacological and nonpharmacological treatment of other CV risk factors (such as obesity, diabetes, and dyslipidemia) will help in blood pressure control and will improve the reduction of CV risk.^7^

### 4.4 Patient empowerment

It is well known that HTN awareness differs substantially around the world. It is more common in high‐income countries than in low‐ or middle‐income countries (67% vs. 38%).^38^ A broader people‐centered model empowering a larger group, envisaging roles not only for patients but also for families, networks and society, should be seen as a possible way to improve adherence to self‐care and therapeutic options in the management of HTN.^5^

Patient and carer education is an important strategy to improve HTN management in primary care settings.^5^ Patients should be empowered to know their BP targets to encourage active engagement in management goal setting. There is strong evidence that self‐monitoring BP, especially as part of a multifaceted intervention (including counseling, telephone support, or telemonitoring), can lead to better treatment adherence and improve BP control.^39^, ^40^ The TASMINH4 and TASMIN‐SR studies have shown that self‐monitoring and self‐titration of antihypertensive medication, following adequate patients' education, resulted in better BP control rates than the usual care.^41^

### 4.5 New technologies

Advances in health technology provide also new opportunities for improving hypertensive care delivery based on digital health and telemedicine.^5^ One step beyond self‐monitoring BP is telemonitoring, which allows patients to obtain their BP measurements at home and transmit readings electronically to the clinical care team, who can transmit back recommendations for lifestyle changes, medication adjustments, or schedule an appointment. This feedback loop could enhance patient engagement in care, avoid unnecessary office visits, and facilitate HTN management, but depends on the secure data‐sharing systems across clinical and community.^42^ WHO recommends the encouragement of both home‐based self‐care and telemonitoring to enhance BP control as part of an integrated management system.^27^ The HOME BP and HERB‐DH1 trials showed that digital application (with interactive education, digital support for nonpharmacological interventions and aids for self‐planning and evaluation) significantly reduced the systolic BP in 3.4 (HOME BP)/3.6 (HERB‐DH1) mmHg, showing not only the feasibility but also the efficacy and low incremental cost of new technologies.^43^

## 5 CONCLUSION

HTN represents a major preventable cardiovascular risk factor. Effective and well‐tolerated antihypertensive agents have been developed over the years, However, suboptimal BP control is still a major problem globally, particularly in low and middle incoming settings, responsible for high morbidity and mortality globally. The identification of barriers and solutions to improve HTN prevention, diagnosis and control differ according to the region and should be tailored to each setting. Those solutions should include strategies to promote education and healthy lifestyle on an individual and community basis, expansion of the diagnostic capacity by the implementation of opportunist screening and out‐off‐office BP measurements, empowerment of patients, maximizing adherence using single‐pill medications, strengthening of primary care, organizing multidisciplinary teams for CV risk prevention, and integrating nonphysician health‐workers on task‐sharing of treatment and management of HTN. The widespread of health technology provides new opportunities for improving HTN prevention and care for both patients and health system levels. This is the time to use all the opportunities to tackle one of the main responsible of global mortality.
